# Do Children Who Were Preschool Picky Eaters Eat Different Foods at School Lunch When Aged 13 Years Than Their Non‐Picky Peers?

**DOI:** 10.1111/jhn.70063

**Published:** 2025-05-14

**Authors:** A. Kemp, P. M. Emmett, C. M. Taylor

**Affiliations:** ^1^ Centre for Academic Child Health, Bristol Medical School University of Bristol Bristol UK

**Keywords:** ALSPAC, child, fussy eating, packed lunch, picky eating, school dinner, school lunch

## Abstract

**Background:**

Picky eating behaviour is characterised by an unwillingness to eat familiar foods, try new foods, and/or strong food preferences. Prevalence peaks at about 3 years of age and usually declines during school years but behavioural characteristics may persist. Parental pressure may influence this. Our aim was to assess food choices in a school setting, away from the family environment, of 13‐year‐old children who were preschool picky eaters compared with those who were never picky eaters.

**Methods:**

Children were recruited at birth in south‐west England and followed to age 13 years. Children (*n* = 7554) were classified as never (26%), low (59%) or high picky eaters (15%) based on parental responses to questionnaires completed when they were pre‐schoolers. In a questionnaire completed at age 13 years (*n* = 5348) the children were asked about frequency of consumption of lunchtime food items at school. Adjusted binary logistic regression modelling was used to determine the associations with picky eating classification.

**Results:**

There were no differences between picky and non‐picky eaters in the frequency of packed lunch compared with school dinner uptake. Both high and low picky eaters were less likely to have meat, fish or cheese/egg sandwiches, or fruit or salad in packed lunches than non‐picky eaters. High picky eaters ate fewer ham/meat sandwiches (OR 0.49 [95% CI 0.39, 0.61]) and fruits (0.62 [0.49, 0.80]) than non‐picky eaters. Picky eaters were more likely to choose meat products in school dinners (e.g. meat burgers/sausages 1.29 [1.06, 1.57]) and have similar intakes of roast meats and fruit, but were less likely to have cooked vegetables or salad (0.68 [0.52, 0.90] and 0.62 [0.46, 0.83] respectively) than non‐picky eaters.

**Conclusion:**

Behaviours characteristic of picky eating, such as avoiding meat, fish and fruit, were less evident in school dinner than packed lunch choices. However, avoidance of vegetables/salad persisted. It is likely that family norms have a stronger influence over packed lunch content than over school dinner choices where the child has more autonomy and may be influenced by their peers.

## Summary

- Prevalence of picky eating in preschool years peaks at about 3 years of age and usually declines during school years but some behavioural characteristics may persist.
- In both packed lunches and school dinners salad and vegetables were more likely to be avoided by children who had been categorised as a picky eater in preschool years.
- Picky children were more likely than non‐picky children to avoid meat, fish and fruit in their packed lunches whereas in school dinners meat, fish and fruit were not eaten less often.
- Family norms may have a stronger influence over packed lunch content than over school dinner choices where the child has more autonomy and may be influenced by their peers.

## 1 Introduction

The definition of picky eating is generally accepted as encompassing sometimes refusing familiar foods, not trying new or unfamiliar foods (food neophobia) and may include slow eating, rejection of foods with certain textures and low enjoyment of food [1, 2, 3].

The prevalence of picky eating ranges from 6% to 50%, depending on the definition chosen by the study, the classification tool, the age of the participants and the culture of the study country [2]. Most research focuses on picky eating behaviour in pre‐pubescent children, and particularly preschool children, as prevalence is greatest in this age group [1, 4, 5, 6]. Although picky eating behaviours tend to decline with age, some can still be detected into adolescence [7] and sometimes into adulthood [8].

As the preschool child develops [9], they have increasing autonomy over the foods that they choose to eat, initially by using basic language and reaching for foods. This can result in developmentally normative and short‐lived picky eating behaviour, which can sometimes persist. This pickiness can be a significant cause of frustration, stress and anxiety to the caregivers, causing conflict and employment of directive strategies such as authoritarian parenting, rewards for eating and pressurising the child to eat [10, 11]. Such strategies can cause further stress for both the child and caregiver at mealtimes and are unlikely to result in positive responses to unfamiliar foods, as well as having a detrimental effect on caregiver–child relationships [12, 13]. Non‐directive strategies such as inclusive family meals, responsive parenting and involvement of the child in meal preparation have instead been shown to increase dietary variety and may contribute to averting longer‐term picky eating behaviour.

The impact of picky eating behaviour can be evidenced empirically in dietary intakes, with a smaller dietary variety and reduced intake of vegetables, fruit and meat in preschool years [7, 14, 15, 16, 17, 18, 19, 20, 21, 22]. Adolescence marks a further stage of developing autonomy. During this period, children may have more opportunities to eat away from the home without the caregivers' supervision. However, to our knowledge, no studies have investigated whether picky eating behaviours persist at times when the children are away from the caregiver's influence and family setting.

Previous studies from the Avon Longitudinal Study of Parents and Children (ALSPAC) have used food records from children at age 3, 7, 10 and 13 years of age and compared food intakes between children with and without picky eating behaviours at preschool ages [7, 16]. These data have shown lower intakes of fruit, vegetables and meat in children who had been picky compared with those who were never picky eaters [7, 16]. The differences lessened as the children grew older [7]. However, the dietary data used in these studies were collected either directly from parents or with considerable parental influence. The investigation of food choices made away from the family such as in a school setting may find a greater influence of peers on the diets of picky children.

This study aims to investigate the school setting. A few months before providing the food record at the age of 13 years, children were sent a questionnaire about foods they were eating away from home to complete themselves and return by post. This covered their consumption of packed lunches and school dinners, and the food items chosen for each as well as consumption of items bought outside school and from vending machines. Therefore the aims of this study were (1) to determine if there are any differences in the frequencies of having packed lunches versus school dinners in 13‐year‐old children who had been identified as preschool picky eaters compared with their non‐picky peers; (2) to determine if there were any differences in the choice of food items within packed lunches and school dinners or foods purchased outside of school between these groups of children.

## 2 Methods

ALSPAC is a large prospective observational study, established to explore environmental and genetic factors affecting a person's health and development. All pregnant women resident in Avon, UK with expected dates of delivery between 1 April 1991 and 31 December 1992 were invited to take part in the study. Of the 20,248 pregnancies identified as being eligible and the number enrolled was 14,541 resulting in 14,062 live births and 13,988 children alive at 1 year of age. Data were collected by self‐completion postal questionnaire completed by parents about their child up to age 5.5 years and from the children themselves at age 13 years. Further details regarding ALSPAC are available at https://www.bristol.ac.uk/alspac/. The study website contains details of all the data that are available through a fully searchable data dictionary and variable search tool which can be found at http://www.bristol.ac.uk/alspac/researchers/our-data/. [23, 24]

*Exposure: Classification of picky eating status.* Questionnaires were completed by the primary caregiver (typically the mother) when their child was 2, 3, 4.5 and 5.5 years old. Picky eating was classified from responses to the following question at each age:

Question: Does your child have definite likes and dislikes as far as food is concerned?

Answer: No/Yes, quite choosy/Yes, very choosy

**Figure 1:**
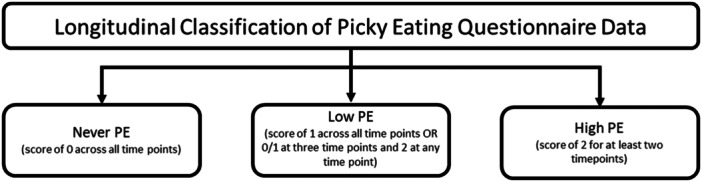
The longitudinal classification of picky eating children in the ALSPAC birth cohort study from ages 2 to 5.5 years. Adapted from Taylor et al. [7].

The answers to this question were scored 0, 1 or 2, respectively, at each age. The longitudinal prevalence of preschool picky eating was categorised as: never (0 at each age), high (2 at ≥ 2 ages) and low (1 at any age or 2 at one age only) (Figure 1) [16].

*Outcomes: Data on school lunches collected via questionnaires.* Children were included in this study if data on their preschool picky eating status were available, and they had answered at least one of three items in a questionnaire on school lunches sent to them when they were about 13 years of age entitled *Food and Things* (http://www.bristol.ac.uk/alspac/researchers/our-data/questionnaires/):

Question: For your lunch in term time, how many times in a week do you:

1. Have a school dinner or buy from the school canteen?
2. Have a packed lunch?
3. Buy food from outside school for lunch?

Answer categories: Never/Once/Twice/4 times/5 times/More than 5 times/Varies

Data regarding their school lunch habits included what foods/drinks, from a list of items, they may have in their packed lunch or chose for school dinner or purchased out of school. The categories available to select were recoded into a binary variable high/low (Low frequency of intake: Never/Once a month or less/Once in 2 weeks; High frequency of intake: Once a week/2–3 times per week/4–5 times per week). For drinks consumption, the variable on the number of cans or bottles of soft drinks consumed per week was categorised as high > 1/low ≥ 1.

*Confounders*

Additional variables were collected from questionnaires completed by the mother in pregnancy. Potential confounders were identified from the literature and included in the demographics analysis. Four variables were selected as possible confounders for use in regression analyses: pre‐pregnancy BMI (kg/m^2^), maternal education (none/CSE/vocational, O level/A level or degree), parity (0 or ≥ 1) and sex of the child. Other variables used in analyses of demographics included maternal age at the birth ( < 25, 25–35, > 35 years), maternal smoking during pregnancy (yes/no), preterm delivery (yes/no), low birthweight (yes/no) and gestational age at birth (weeks).

### 2.1 Statistical Analysis

The analytical plan was pre‐specified and any data‐driven variations are identified. SPSS v28 (IBM Corp.) was used to carry out the statistical analyses.

The characteristics of included participants by picky eating status were compared using chi‐square tests for categorial data or means with standard deviations for continuous data (from one‐way ANOVA analysis). The characteristics of included and excluded participants were similarly compared.

The frequency of having packed lunch, school dinner or eating outside school by picky eating classification was determined and compared with chi‐squared tests. The frequency of eating each food item by picky eating classification under groupings of packed lunch, school dinner or outside school/vending machine, was determined and compared in the same way. The frequencies of consumption of types of drinks were similarly compared.

Adjusted binary logistic regression was used to determine the strength of associations between picky eating status and drink and food item consumption. The ‘non‐picky eaters’ were used as the reference group for all regression analysis.

## 3 Results

### 3.1 Study Participants

Of 15,614 participants who ever attended ALSPAC, 7554 children had data to enable classification of their pre‐school picky eating status: 1974 (26%) were non‐picky eaters, 4478 (59%) were low picky eaters, 1102 (15%) were high picky eaters. Higher maternal educational attainment, lower maternal BMI and being the first‐born child were associated with the child being a high picky eater (Table 1).

**Table 1:** Demographics of 13‐year‐old children and their mothers classified by preschool picky eating status in ALSPAC (*n* = 5348).

Characteristic	*n*	Picky eating classification			*p* value
		Never PE	Low PE	High PE	
Maternal age (years)^a^	5348				0.709
< 25		189 (13.8%)	389 (12.2%)	102 (12.8%)	
25–35		1069 (77.8%)	2504 (78.8%)	624 (78.2%)	
> 35		116 (8.4%)	283 (8.9%)	72 (9.0%)	
Pre‐pregnancy BMI (kg/m^2^)^b^	4989	23.1 (3.7)	22.7 (3.6)	22.7 (3.6)	0.005
Maternal education^a^	5291				< 0.001
None/CSE/Vocational		291 (21.5%)	594 (18.9%)	150 (19.0%)	
O level/A level		872 (64.3%)	1932 (61.4%)	477 (60.5%)	
Degree		193 (14.2%)	620 (19.7%)	162 (20.5%)	
Parity during index pregnancy^a^	5180				0.002
0		590 (44.6%)	1494 (48.5%)	407 (52.5%)	
≥ 1		732 (55.4%)	1589 (51.5%)	368 (47.5%)	
Maternal smoking^a^	5307				0.898
No		1141 (83.8%)	2657 (84.2%)	660 (83.5%)	
Yes		220 (16.2%)	500 (15.8%)	130 (16.5%)	
Child's sex^a^	5348				0.114
Male		649 (47.2%)	1462 (46.0%)	400 (50.1%)	
Female		725 (52.8%)	1714 (54.0%)	398 (49.9%)	
Preterm birth (< 37 weeks)^a^	5348				0.402
Yes		76 (5.5%)	148 (4.7%)	36 (4.5%)	
No		1298 (94.5%)	3028 (95.3%)	762 (95.5%)	
Low birth weight (< 2500 g)^a^	5283				0.716
Yes		51 (3.7%)	123 (3.9%)	35 (4.4%)	
No		1311 (96.3%)	3011 (96.1%)	752 (95.6%)	
Gestational length (weeks)	5348	39.5 (1.9)	39.5 (1.8)	39.5 (1.7)	0.178

*Note:* Picky eaters aged between 2 and 5.5 years were classified as either non‐picky, low or high picky eaters.

Abbreviations: BMI, body mass index; CSE, certificate of secondary education; PE, picky eater.

Total *n* for each demographic is different due to missing data and participants being able to withdraw consent for some information being accessible.

^a^ Statistical tests used: x‐tab chi‐square test.

^b^ Statistical tests used: mean and standard deviation *t*‐tests (one‐way ANOVA).

**Figure 2:**
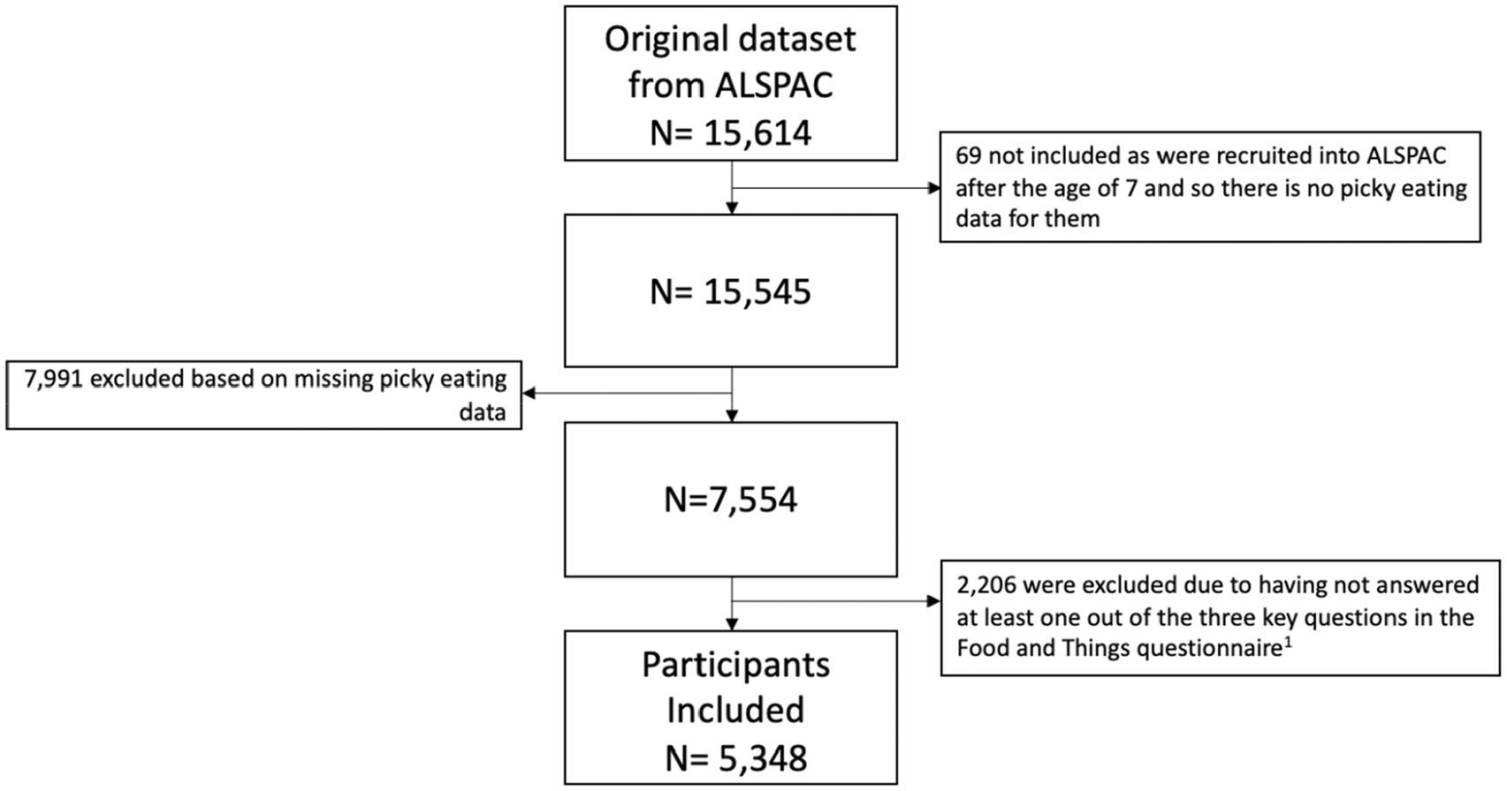
Flow diagram outlining the exclusion process from the complete data set of participants in ALSPAC. ^1^The three key questions from the Food and Things questionnaire are as follows: Question: For your lunch in term time how many times in a week do you: Have a school dinner or buy from the school canteen. Have a packed lunch. Buy food from outside school for lunch. Answer: Never/Once/Twice/4 times/5 times/More than 5 times.

Children with known picky eating status were excluded if they had not answered at least one of the three questions on lunchtime eating at age 13 years (*n* = 2206), leaving 5348 participants for further analysis (Figure 2).

Table S1 outlines the differences between the included and excluded participants. The included sample differed from those excluded in several ways: their mothers were likely to be older, have lower BMI, have higher educational attainment and have never smoked; the children were less likely to be preterm or have a low birth weight, have a higher gestational age at birth, were more likely to be first born and were more often female.

About half of the included children had packed lunch on most days and a further quarter on some days (Table 2). Slightly more than a quarter had school dinners on most days with 38% having them on some days. Most children never ate outside of school at lunch time (87%) and a quarter of children admitted to sometimes skipping lunch completely. The frequencies of having packed lunches, or school dinners, or buying lunch outside of school or skipping lunch completely were not associated with picky eating status.

**Table 2:** Frequency children in the ALSPAC birth cohort had packed lunches, school dinners and ate outside of school at age 13 years in relation to their preschool picky eating status between ages 2 and 5.5 years of age (total *n* = 5348).

		*n*	Picky eating classification			*p* value (chi‐squared test)
			Never PE	Low PE	High PE	
Packed lunch	Never	5319	325 (23.7%)	795 (25.2%)	210 (26.6%)	0.590
	1–3 times		335 (24.5%)	729 (23.1%)	180 (22.8%)	
	4–5+ times		709 (51.8%)	1636 (51.8%)	400 (50.6%)	
School dinner	Never	5332	472 (34.5%)	1092 (34.5%)	273 (34.3%)	0.947
	1–3 times		528 (38.6%)	1190 (37.6%)	299 (37.6%)	
	4–5+ times		368 (26.9%)	887 (28.0%)	223 (28.1%)	
Outside school	Never	4240	1110 (87.0%)	2567 (87.7%)	645 (87.7%)	0.798
	Ever		166 (13.0%)	359 (12.3%)	93 (12.6%)	
Frequency on school days child misses lunch completely	Never	5139	972 (72.3%)	2278 (74.7%)	600 (77.1%)	0.121
	Ever		227 (16.9%)	451 (14.8%)	115 (14.8%)	
	Varies		144 (10.7%)	319 (10.5%)	63 (8.1%)	

*Note:* Total *n* for each category is different due to participants being able to choose to not answer questions in the *Food and Things* questionnaire.

Abbreviation: PE, picky eater.

### 3.2 Packed Lunches

An average of 72% of the cohort answered the questions on packed lunches. The most popular sandwich fillings were meat‐ and fish‐based. Many children (78%) had crisps or alternatives but less than half had salad at least once a week in their packed lunches. Fruit (75%) and chocolate bars (77%) were popular. There were many differences in lunch choices between the picky eating groups.

The adjusted regression analysis (Table 3) showed that picky children were much less likely than non‐picky children to have ham/meat or tuna/fish in their sandwiches, with cheese/egg fillings also being avoided but to a slightly lesser extent (see Table S2 for univariate analysis). Very picky children were 46% more likely to have marmite, peanut butter or cheese spread instead (*p* < 0.001). Crisps or alternatives were included slightly less often by very picky children (*p* = 0.004). Picky children were much less likely to include salad than non‐picky children (low 25% less, high 58% less (both *p* < 0.001)). High picky children had fruit 38% less often than non‐picky children. The frequency of other sweet foods was not different by picky eating status.

**Table 3:** Adjusted binary logistic regression analysis of the packed lunch food group choices of children at 13 years of age by preschool picky eating status.

Packed lunch choice	Never PE: % eating ≥ once/week)	Regression analysis			
			*n*	OR (95% CI)	*p* value
Sandwiches					
Ham/meat	71.4	Low PE	3481	0.75 (0.63, 0.89)	< 0.001
		High PE		0.49 (0.39, 0.61)	0.001
Cheese/egg	42.1	Low PE	3456	0.87 (0.74, 1.02)	0.096
		High PE		0.64 (0.51, 0.81)	< 0.001
Tuna/fish	58.8	Low PE	3457	0.66 (0.56, 0.78)	< 0.001
		High PE		0.29 (0.23, 0.37)	< 0.001
Marmite/peanut butter/cheese spread	18.9	Low PE	3454	1.09 (0.89, 1.32)	0.410
		High PE		1.46 (1.13, 1.89)	< 0.001
Jam/honey/chocolate spread	33.4	Low PE	3448	0.78 (0.66, 0.92)	0.004
		High PE		0.84 (0.67, 1.07)	0.155
Pies/pasties	9.6	Low PE	3481	0.99 (0.75, 1.30)	0.986
		High PE		0.68 (0.45, 1.04)	0.683
Crisps/corn snacks/wotsits	78.3	Low PE	3499	0.84 (0.70, 1.02)	0.078
		High PE		0.76 (0.59, 0.99)	0.039
Lunchables	3.5	Low PE	3427	1.06 (0.68, 1.66)	0.793
		High PE		1.18 (0.65, 2.14)	0.583
Cheese strings/Baby Bel	21.3	Low PE	3463	0.80 (0.66, 0.97)	0.023
		High PE		0.90 (0.69, 1.18)	0.453
Peperami	9.1	Low PE	3447	0.73 (0.55, 0.97)	0.029
		High PE		0.98 (0.67, 1.43)	0.908
Fruit	75.0	Low PE	3499	0.85 (0.71, 1.01)	0.079
		High PE		0.62 (0.49, 0.80)	< 0.001
Salad	45.0	Low PE	3484	0.75 (0.64, 0.89)	< 0.001
		High PE		0.42 (0.33, 0.53)	< 0.001
Yoghurt/Fromage Frais	42.1	Low PE	3487	1.07 (0.92, 1.26)	0.380
		High PE		0.98 (0.78, 1.22)	0.824
Chocolate/chocolate bars	77.1	Low PE	3510	1.09 (0.90, 1.31)	0.386
		High PE		1.16 (0.89, 1.51)	0.264
Cake	24.3	Low PE	3490	1.05 (0.87, 1.26)	0.618
		High PE		0.87 (0.67, 1.12)	0.282

*Note:* Reference category: Never picky eater. Adjustments were made for maternal pre‐pregnancy body mass index (kg/m^2^), maternal education (none/CSE/vocational, O level/A level or degree), pregnancy parity (0 or ≥ 1) and sex. *n* values differ due to missing information on the covariates. Odds ratios (OR) for: Low frequency of intake (reference) (never/once a month or less/once in 2 weeks) vs High frequency of intake (Once a week/2–3 times per week/4–5 times per week).

### 3.3 School Dinners

The proportion of the cohort providing information on school dinner items averaged 61% (Table S2). The most popular main courses, once a week or more, were pizza/lasagne/pasta bake (74%) and sandwiches (69%). Cakes/buns/biscuits/cookies (70%) were the most popular sweet course. Cooked vegetables, salad and fruit were each chosen by only one quarter of children. There were fewer differences in school dinners by picky eating status than for packed lunches.

The adjusted regression analysis confirmed that very picky children were 49% more likely than non‐picky children to choose burgers/sausages for their cooked lunch (Table 4). However, most other cooked mains were not different by picky eating status, except that stew/curry/bolognaise and pizza/lasagne/pasta bake were chosen slightly less often by picky eaters. Sandwiches from the canteen were also slightly less likely to be chosen by picky eaters. The most consistent difference between the groups was that the very picky children chose salad/coleslaw/raw vegetables (38%) and cooked vegetables (32%) less often than the non‐picky children. Most of the sweet foods including fruit were not different, but yoghurt/fromage frais was chosen less often by low (34% less) and high picky eaters (30%) and mousse/trifle was chosen 38% less often by low picky eaters.

**Table 4:** Adjusted binary logistic regression analysis of the school dinner food group choices of children at 13 years of age by preschool picky eating status.

School dinner choices	Never PE: % eating ≥ once/week)	Regression analysis			
			*n*	OR (95% CI)	*p* value
Meat burgers/sausages	26.3	Low PE	2921	1.29 (1.06, 1.57)	0.010
		High PE		1.49 (1.15, 1.93)	0.003
Meat pies/sausage rolls	15.3	Low PE	2903	1.13 (0.89, 1.44)	0.313
		High PE		1.35 (0.99, 1.86)	0.060
Vegetarian pies/sausages/samosas	4.3	Low PE	2892	1.04 (0.68, 1.58)	0.870
		High PE		0.84 (0.46, 1.54)	0.576
Stew, curry, bolognaise	23.8	Low PE	2892	0.90 (0.73, 1.10)	0.287
		High PE		0.73 (0.55, 0.98)	0.037
Roast meat	22.3	Low PE	2898	0.84 (0.68, 1.04)	0.115
		High PE		0.85 (0.64, 1.14)	0.289
Eggs, quiche	6.4	Low PE	2894	0.77 (0.53, 1.11)	0.162
		High PE		0.76 (0.45, 1.27)	0.292
Fish/fish fingers	18.2	Low PE	2898	0.91 (0.72, 1.14)	0.389
		High PE		0.93 (0.68, 1.27)	0.650
Baked beans/spaghetti	28.1	Low PE	2898	0.92 (0.76, 1.12)	0.400
		High PE		0.85 (0.65, 1.12)	0.249
Pizza/lasagne/pasta bake	73.9	Low PE	2915	0.81 (0.66, 0.98)	0.030
		High PE		0.84 (0.64, 1.10)	0.200
Sandwiches from the canteen	68.6	Low PE	2901	0.83 (0.69, 0.99)	0.043
		High PE		0.79 (0.62, 1.02)	0.070
Chips/roast potatoes/croquettes	47.5	Low PE	2903	1.10 (0.92, 1.30)	0.307
		High PE		1.18 (0.93, 1.49)	0.174
Other potatoes/rice	30.2	Low PE	2889	0.98 (0.81, 1.18)	0.792
		High PE		0.85 (0.65, 1.11)	0.851
Cooked vegetables	30.3	Low PE	2882	0.84 (0.69, 1.01)	0.069
		High PE		0.68 (0.52, 0.90)	0.006
Salad/coleslaw/raw vegetables	27.0	Low PE	2890	0.88 (0.72, 1.07)	0.204
		High PE		0.62 (0.46, 0.83)	0.001
Hot puddings	17.5	Low PE	2883	0.89 (0.70, 1.11)	0.298
		High PE		0.94 (0.68, 1.28)	0.678
Cakes/buns/biscuits/cookies	70.4	Low PE	2890	0.98 (0.81, 1.19)	0.846
		High PE		1.01 (0.78, 1.32)	0.915
Yoghurt/fromage frais	16.4	Low PE	2869	0.66 (0.52, 0.84)	< 0.001
		High PE		0.70 (0.50, 0.98)	0.039
Fruit	24.7	Low PE	2865	0.94 (0.77, 1.16)	0.573
		High PE		0.89 (0.59, 1.05)	0.104
Mousses/mousse pots/trifles	11.2	Low PE	2867	0.62 (0.46, 0.84)	0.002
		High PE		0.82 (0.55, 1.22)	0.321

*Note:* Reference category: Never picky eater. Odds ratios (OR) for: Low frequency of intake (reference) (Never/Once a month or less/Once in two weeks) vs High frequency of intake (Once a week/2–3 times per week/4–5 times per week). Adjustments were made for pre‐pregnancy body mass index (kg/m^2^), maternal education (none/CSE/vocational, O level/A level or degree) and pre‐index pregnancy parity (0 or ≥ 1) and sex of the child. *n* values differ due to missing information on the covariates.

### 3.4 Outside School/Vending Machines (Including After School and Weekends)

Most of the cohort (90%) answered questions on buying and eating foods outside of school or from school vending machines. Since only 13% of the children indicated that they had food outside school at lunch time (Table 1), most of these foods are likely to have been bought at other times. Around half of the children were obtaining sweets/chocolates and one‐third obtaining crisps or alternatives from sources outside of school meals. There were very few differences in foods from outside school by picky eating status (Table S2). Low picky eaters were slightly less likely to buy fruit (19%) or sandwiches (19%) at least once a week than the non‐picky eaters with no difference for very picky eaters (Table 5).

**Table 5:** Adjusted binary logistic regression analysis of outside school or vending machine food group choices of children at 13 years of age by preschool picky eating status.

Outside school or vending machine food group choice	Never PE: % eating ≥ once/week)	Regression analysis			
			*n*	OR (95% CI)	*p* value
Chips	18.8	Low PE	4169	0.91 (0.75–1.10)	0.303
		High PE		1.03 (0.80–1.33)	0.798
Fruit	29.0	Low PE	3096	0.81 (0.69–0.95)	0.011
		High PE		0.87 (0.70–1.09)	0.235
Burgers	7.9	Low PE	4146	0.99 (0.75–1.30)	0.869
		High PE		0.96 (0.66–1.40)	0.918
Sandwiches	22.4	Low PE	4147	0.81 (0.68–0.96)	0.018
		High PE		0.93 (0.73–1.19)	0.571
Pie/pastie	7.4	Low PE	4140	0.96 (0.73–1.28)	0.788
		High PE		0.86 (0.58–1.29)	0.472
Pizza	11.8	Low PE	4139	0.91 (0.72–1.15)	0.440
		High PE		1.12 (0.83–1.53)	0.459
Chocolate/sweets	48.0	Low PE	4215	0.95 (0.82–1.09)	0.422
		High PE		1.04 (0.85–1.27)	0.666
Crisps	33.9	Low PE	4170	0.94 (0.80–1.10)	0.435
		High PE		1.05 (0.85–1.30)	0.644
Other	24.5	Low PE	4137	0.80 (0.68–0.94)	0.008
		High PE		0.89 (0.71–1.11)	0.308

*Note:* Reference category: Never picky eater. Odds ratios (OR) for: Low frequency of intake (reference) (never/once a month or less/once in 2 weeks) vs. High frequency of intake (Once a week/2–3 times per week/4–5 times per week). Adjustments were made for pre‐pregnancy body mass index (kg/m^2^), maternal education (none/CSE/vocational, O level/A level or degree) and pre‐index pregnancy parity (0 or ≥ 1) and sex of the child. *n* values differ due to missing information on the covariates.

### 3.5 Drinks

High picky eaters were 25% less likely to drink pure fruit juice and 34% more likely to drink decaffeinated cola drinks than the non‐picky eaters in adjusted regression analyses (Tables S3 and S4). Both high and low picky eaters were more likely to drink low‐calorie/low‐sugar soft drinks than non‐picky eaters (20% and 25%, respectively), but the associations were weak.

### 3.6 Model Variance

The R‐square value for each model ranged from 0.004 to 0.048 meaning that the maximum variance the models accounted for was just under 5%.

## 4 Discussion

There were no differences in the frequencies of having packed lunches versus school dinners in 13‐year‐old children who had been identified as preschool picky eaters compared with their non‐picky peers. Foods eaten as packed lunches and as school dinners by the picky eaters differed from those of non‐picky eaters. The fully adjusted regression analyses showed that both high and low picky eaters were less likely to have meat, fish or cheese/egg sandwiches in packed lunches and were less likely to eat fruit or salad. For those having school dinners, roast meat intake did not differ, but picky eaters were more likely to have meat products, and they were less likely to have cooked vegetables or salad/coleslaw/raw vegetables. Picky eaters also avoided yoghurt/fromage frais and mousse/trifle more often than non‐picky eaters in school dinners, but in packed lunches, they were eaten equally often by all groups. There were only minimal differences between picky and non‐picky children in foods/drinks purchased outside of school. Our previous study with this group of children in ALSPAC that assessed the overall diet by food record when they were 10 and 13 years old found lower intakes of meat, fruit and vegetables at ages 10 and 13 years in picky eaters than non‐picky eaters [7], but most of the meals recorded were consumed within the family. In this study we focused on school lunches where the child was away from direct familial influence. Here avoidance of meat, fish, fruits and salads was evident in the packed lunches that were likely to have been brought from home. However, there was little evidence of picky eaters avoiding meat, fish and fruit in school dinners where there is no familial influence but in contrast, avoidance of vegetables and salad persisted. Our results suggest that some picky eating behaviours persist while others may be modified in adolescents when they are away from direct familial influence, such as when eating school dinners with their peers.

We have previously shown that picky eating behaviours persist in the whole diet at 13 years of age in this group of children [7]. Similarly, a study in the Netherlands has shown that picky eating originally assessed at 4 years of age can persist into early adulthood (17–20 years) [25].

Our results on the avoidance by picky eaters of vegetables and salad are consistent with findings from other studies, as picky eaters having reduced vegetable intake is a well‐documented association [16, 17, 18, 19, 26, 27]. Vegetables are noted by some researchers to be the most frequently avoided food group in picky eaters [14, 26, 27, 28, 29, 30, 31]. It is likely that the avoidance of vegetables and salads by the picky eaters identified as preschoolers and now 13 years of age has become an integral part of their eating style, while our results suggest that eating with their peers may have widened their intake of meat, fish and fruit.

Picky eaters were more likely than non‐picky eaters to have meat burgers or sausages in school dinners while avoiding cold meat in packed lunch sandwiches. Picky eaters have been shown to be sensitive to the temperature of foods [32] – they tend to prefer warm foods over cold – and so this may be a factor affecting these choices.

In this study, picky eaters were consuming less variety of foods in their packed lunches than non‐picky eaters, and it is likely therefore that the nutritional quality of their meals was compromised. In a Canadian study comparing nutrient intakes from packed lunches between picky eaters and non‐picky eaters aged 7–10 years, picky eaters had lower nutrient intakes from the meal than non‐picky eaters [32]. Previous work in ALSPAC has shown that the nutritional quality of packed lunches tends to be worse than that of school dinners [33]. Thus, it is particularly important that the parents of preschool picky eaters are helped to ensure that their children's packed lunches are as nutritious as possible in accordance with national guidance on nutrition [34].

To our knowledge, picky eaters choosing to have less yoghurt/fromage frais or mousse/trifle than non‐picky eaters has not been noted before. This may be due to the texture of these foods: picky eaters can be selective over the texture of foods, with ‘slimy’ or ‘mushy’ foods being among some of the most unappealing textures [32], possibly driven by disgust sensitivity [35].

Our results focused on meals eaten at school, a location in which the immediate familial influence is absent. Authoritarian parenting was identified in a scoping review as an extrinsic factor that increases the likelihood of picky eating [10]. Parenting styles have been found to be highly related to food refusal behaviours, with a more coercive style associated specifically with food fussiness and food neophobia [36]. Parent–child interaction has usually been regarded as uni‐directional, with expression of child autonomy, in this case around food choices, being regarded as noncompliant behaviour. More recently, it has been argued that picky eating should be re‐framed as eating preferences in response to child agency, rather than categorised as compliant versus non‐compliant behaviour [37]. This is reinforced by a study showing that fewer fussy behaviours were expressed when parents provided structured mealtimes but children were allowed some autonomy over food choice and intake [38]. Although the children in the present study were beyond the accepted peak age of picky eating in this cohort (about 3 years old) [2] and outside the family environment, it was evident that picky eating behaviours were still being expressed. This could lead to concern about the development of eating disorders in adolescence and of adult picky eating behaviours. Picky eating has been identified as a risk factor for subsequent anorexia nervosa in one observational study [39], although in a later extension of that study picky eating was not found to be associated with later anorexia or bulimia [40], making interpretation difficult. Adult picky eating has been shown to be associated with parental feeding practices in childhood (particularly pressure to eat) and childhood picky eating [41].

There are several strengths to the design of our study. First, the questionnaire about school lunches was completed in relatively large numbers by the children themselves. This was important for our study as, unlike other studies that rely on parental perception of their child's picky eating behaviours, having the children report their own food intake gave us a reflection of their eating habits away from parental interpretation. In addition, non‐picky eater comparison groups were included for all analyses. The longitudinal nature of our data enabled us to assess picky eating behaviours over time.

There are also several limitations. While it is likely that a caregiver was responsible for the foods available in the home from which to select the packed lunch items, we do not know who decided which items to include. It is likely that foods included in a packed lunch are influenced by family norms at least to some extent. Food safety may be a concern for parents when making their child's lunch [42] due to lack of refrigerated storage in schools, this may restrict the food options parents are willing to provide [42], although this would apply equally to picky eaters and non‐picky eaters. School dinner choices are more obviously free from parental influence, but the choice of items is set by the school, or the caterer, so is limited to some extent. Children in lower socioeconomic groups are more likely to have school dinners if they qualify for free school meals and we did not have information about this available to the study. However, there was no difference between the picky eating groups in the take up of school dinners.

As seen in Table S1, the included group of participants in this study is somewhat different from the excluded group, introducing potential selection bias as our included group is not representative of the original ALSPAC population. There may be additional variables that we were unable to account for in our models.

Picky eating status in ALSPAC was assessed over four pre‐school ages via a single question based on parental perception of how choosy their child was with food at each age. In this method, the child must be picky at more than one age. At the time picky eating was assessed in ALSPAC, there was no gold standard tool, as the questionnaires that are now frequently used had not been developed and validated. The method used here has been shown to reliably differentiate picky eaters from non‐picky eaters in other studies [7, 16, 26]. However, it did not cover a full range of picky eating behaviours such as textures, smells, eating contexts and frequency of food rejection.

## 5 Conclusion

In conclusion, children who had been preschool picky eaters were as likely as their non‐picky peers to have packed lunches or school dinners when they were adolescents aged 13 years. However, the food groups eaten during these meals varied between picky and non‐picky eaters. Picky children were more likely than non‐picky children to avoid meat, fish and fruit in their packed lunches, whereas in school dinners meat, fish and fruit were not eaten less often. However, in both packed lunches and school dinners salad and vegetables were more likely to be avoided by picky eaters: this is a well‐documented picky eating behaviour. It is likely that family norms have a stronger influence over packed lunch content than over school dinner choices, where the child has more autonomy and may be influenced by their peers.

## Author Contributions

C. M. Taylor and P. M. Emmett designed the study with input from A. Kemp. A. Kemp performed the analyses with guidance from C. M. Taylor and P. M. Emmett. A. Kemp wrote the manuscript with critical input and revisions from C. M. Taylor and P. M. Emmett. All authors approved the final version of the manuscript.

## Ethics Statement

Ethics approval for the study was obtained from the ALSPAC Ethics and Law Committee and the Local Research Ethics Committees (http://www.bristol.ac.uk/alspac/researchers/research-ethics/). Informed consent for the use of data collected via questionnaires and clinics was obtained from participants following the recommendations of the ALSPAC Ethics and Law Committee at the time.

## Conflicts of Interest

The authors declare no conflicts of interest.

### 1 Peer Review

The peer review history for this article is available at https://www.webofscience.com/api/gateway/wos/peer-review/10.1111/jhn.70063.

## Transparency Declaration

The lead author affirms that this manuscript is an honest, accurate and transparent account of the study being reported. The reporting of this study is compliant with STROBE guidelines. The lead author affirms that no important aspects of the study have been omitted and that any discrepancies from the study as planned have been explained.

## Supporting information

JHND Picky eating School lunches Suppl Mat.

## Data Availability

The ALSPAC study website contains details of all the data that are available through a fully searchable data dictionary and variable search tool which can be found at http://www.bristol.ac.uk/alspac/researchers/our-data/.
