# Supplementary material for: Do Children Who Were Preschool Picky Eaters Eat Different Foods at School Lunch When Aged 13 Years Than Their Non‐Picky Peers?
Source: J Hum Nutr Diet. 2025 May 14;38(3):e70063. doi: 10.1111/jhn.70063 (PMC12077986; doi:10.1111/jhn.70063)
Supplement: Supplementary file 1 — JHND Picky eating School lunches Suppl Mat. [file JHN-38-0-s001.docx]

**Supplementary material**

**Supplementary Table 1**Comparing demographic characteristics for those included and excluded from this study (total included n=5348 from full ALSPAC dataset of 15,614)

| **Characteristic** | **Included (34.2%)** | | **Excluded (65.8%)** | | **p value** |
| --- | --- | --- | --- | --- | --- |
|  | **n** | **%** | **n** | **%** |  |
| Maternal age (years)^a^ |  |  |  |  |  |
| <25 | 5348 | 680 (12.7%) | 8704 | 2681 (30.8%) | <0.001 |
| 25-35 |  | 4197 (78.5%) |  | 5519 (63.4%) |  |
| >35 |  | 471 (8.8%) |  | 504 (5.8%) |  |
| Pre-pregnancy BMI (kg/m^2^)^b^ | 4989 | 22.8 ± 3.6 | 6657 | 23.0 ± 4.0 | 0.007 |
| Maternal education^a^ | 5291 |  |  |  |  |
| None/CSE/Vocational |  | 1035 (19.6%) | 7287 | 2826 (38.8%) | <0.001 |
| O level/A level |  | 3281 (62.0%) |  | 3830 (52.6%) |  |
| Degree |  | 975 (18.4%) |  | 631 (8.7%) |  |
| Parity^a^ | 5180 |  |  |  |  |
| 0 |  | 2491 (48.1%) | 7736 | 3328 (43.0%) | <0.001 |
| ≥1 |  | 2689 (51.9%) |  | 4408 (57.0%) |  |
| Maternal smoking^a^ | 5307 |  |  |  |  |
| Never smoked |  | 4457 (84.0%) | 8029 | 5516 (68.7%) | <0.001 |
| Smoked |  | 850 (16.0%) |  | 2513 (31.3%) |  |
| Maternal social class^a^ | 4619 |  | 5481 |  |  |
| I and II |  | 2041 (44.2%) |  | 1732 (31.6%) | <0.001 |
| III |  | 2177 (47.1%) |  | 2933 (53.5%) |  |
| IV and V |  | 401 (8.7%) |  | 816 (14.9%) |  |
| Child’s sex^a^ | 5348 |  | 9682 |  |  |
| Male |  | 2511 (47.0%) |  | 5173 (53.4%) | <0.001 |
| Female |  | 2837 (53.0%) |  | 4509 (46.6%) |  |
| Gestational age (weeks)^b^ | 5348 | 39.5 ± 1.8 | 9241 | 37.7 ± 6.7 | <0.001 |
| Preterm birth (<37 weeks)^a^ | 348 |  | 9241 |  | <0.001 |
| Yes |  | 260 (4.9%) |  | 1175 (12.7%) |  |
| No |  | 5088 (95.1%) |  | 8066 (87.3%) |  |
| Low birth weight (<2500g)^a^ | 5283 |  | 8588 |  | <0.001 |
| Yes |  | 209 (4.0%) |  | 602 (7.0%) |  |
| No |  | 5074 (96.0%) |  | 7986 (93.0%) |  |

BMI, body mass index; CSE, certificate of secondary education

Statistical tests used: ^a^x-tab chi-square test; ^b^mean and standard deviation t-test (one-way ANOVA)

Total n for each demographic is different due to missing data and participants being able to withdraw consent for some information being accessible

**Supplementary Table 2**Food item frequency in packed lunches, school dinners and outside of school for children in the ALSPAC birth cohort study at age 13 years based on longitudinal picky eating classification between ages 2 and 5.5 years (total n=5348)

|  | **Frequency of intake** | **n** | **Picky eating classification** | | | **P value** |
| --- | --- | --- | --- | --- | --- | --- |
|  |  |  | **Never PE** | **Low PE** | **High PE** |  |
| **Packed lunch** | | | | | | |
| Meat/ham | Low | 3884 | 291 (28.6%) | 804 (35.0%) | 251 (44.0%) | <0.001 |
|  | High |  | 728 (71.4%) | 1491 (65.0%) | 319 (56.0%) |  |
| Cheese/egg | Low | 3853 | 584 (57.8%) | 1370 (60.1%) | 377 (67.1%) | <0.001 |
|  | High |  | 426 (42.1%) | 911 (39.9%) | 185 (32.9%) |  |
| Tuna/fish | Low | 3857 | 418 (41.2%) | 1147 (50.2%) | 394 (70.7%) | <0.001 |
|  | High |  | 596 (58.8%) | 1139 (49.8%) | 163 (29.3%) |  |
| Marmite/peanut butter/cheese spread | Low | 3853 | 820 (81.1%) | 1775 (77.8%) | 407 (72.7%) | <0.001 |
|  | High |  | 191 (18.9%) | 507 (22.2%) | 153 (27.4%) |  |
| Jam/honey/chocolate spread | Low | 3847 | 671 (66.6%) | 1606 (70.5%) | 387 (68.9%) | 0.084 |
|  | High |  | 336 (33.4%) | 672 (29.5%) | 175 (31.1%) |  |
| Crisps/corn snacks/Wotsits | Low | 3903 | 221 (21.6%) | 561 (24.3%) | 141 (24.8%) | 0.004 |
|  | High |  | 800 (78.3%) | 1752 (75.7%) | 428 (75.2%) |  |
| Chocolate/chocolate bars | Low | 3912 | 235 (23.0%) | 523 (22.5%) | 120 (21.1%) | 0.674 |
|  | High |  | 787 (77.1%) | 1798 (77.4%) | 449 (78.9%) |  |
| Fruit | Low | 3902 | 256 (25.0%) | 625 (27.0%) | 192 (34.0%) | 0.003 |
|  | High |  | 766 (75.0%) | 1690 (73.0%) | 373 (66.0%) |  |
| Salad | Low | 3880 | 557 (55.0%) | 1372 (59.6%) | 413 (73.2%) | <0.001 |
|  | High |  | 456 (45.0%) | 931 (40.4%) | 151 (26.7%) |  |
| Lunchables | Low | 3822 | 967 (96.5%) | 2179 (96.3%) | 535 (96.1%) | 0.897 |
|  | High |  | 35 (3.5%) | 84 (3.7%) | 22 (3.9%) |  |
| Cheese strings/Baby Bel | Low | 3863 | 797 (78.7%) | 1875 (81.9%) | 450 (80.2%) | 0.086 |
|  | High |  | 216 (21.3%) | 414 (18.1%) | 111 (19.8%) |  |
| Pepperami | Low | 3844 | 915 (90.9%) | 2116 (92.9%) | 508 (90.7%) | 0.057 |
|  | High |  | 92 (9.1%) | 161 (7.1%) | 52 (9.3%) |  |
| Yogurt/fromage fresh | Low | 3891 | 587 (57.9%) | 1280 (55.4%) | 330 (58.3%) |  |
|  | High |  | 427 (42.1%) | 1031 (44.6%) | 236 (41.7%) |  |
| Pie or pasties | Low | 3879 | 915 (90.4%) | 2087 (90.6%) | 523 (92.9%) | 0.195 |
|  | High |  | 97 (9.6%) | 217 (9.4%) | 40 (7.1%) |  |
| Cake | Low | 3889 | 771 (75.7%) | 1708 (74.1%) | 431 (76.3%) | 0.409 |
|  | High |  | 247 (24.3%) | 598 (24.9%) | 134 (23.7%) |  |
| **School dinner** | | | | | | |
| Meat burgers/sausages | Low | 3253 | 610 (73.7%) | 1324 (68.6%) | 326 (66.0%) | 0.005 |
|  | High |  | 218 (26.3%) | 607 (31.4%) | 168 (34.0%) |  |
| Meat pies/sausage rolls | Low | 3230 | 699 (84.7%) | 1599 (83.5%) | 396 (80.7%) | 0.153 |
|  | High |  | 126 (15.3%) | 315 (16.5%) | 95 (19.3%) |  |
| Vegetarian pies/sausages/samosas | Low | 3321 | 787 (95.7%) | 1820 (95.4%) | 474 (96.3%) | 0.674 |
|  | High |  | 35 (4.3%) | 87 (4.6%) | 18 (3.7%) |  |
| Stew, curry, bolognaise | Low | 3238 | 630 (76.2%) | 1479 (77.1%) | 402 (81.7%) | 0.049 |
|  | High |  | 197 (23.8%) | 440 (22.9%) | 90 (18.3%) |  |
| Roast meat | Low | 3226 | 641 (77.7%) | 1530 (80.1%) | 394 (80.2%) | 0.326 |
|  | High |  | 184 (22.3%) | 380 (19.9%) | 97 (19.8%) |  |
| Eggs, quiche | Low | 3220 | 772 (93.6% | 1807 (94.9%) | 469 (95.5%) | 0.239 |
|  | High |  | 53 (6.4%) | 97 (5.1%) | 22 (4.5%) |  |
| Fish/fish fingers | Low | 3224 | 675 (81.8%) | 1584 (83.0%) | 408 (83.3%) | 0.719 |
|  | High |  | 150 (18.2%) | 325 (17.0%) | 82 (16.7%) |  |
| Baked beans/spaghetti | Low | 3227 | 595 (71.9%) | 1395 (73.0% | 366 (74.8%) | 0.519 |
|  | High |  | 232 (28.1% | 516 (27.0% | 123 (25.2% |  |
| Pizza/lasagna/pasta bake | Low | 3243 | 216 (26.1%) | 565 (29.4%) | 147 (29.8%) | 0.181 |
|  | High |  | 611 (73.9%) | 1357 (70.6%) | 347 (70.2%) |  |
| Sandwiches from canteen | Low | 3226 | 259 (31.4%) | 720 (37.7%) | 214 (43.6%) | <0.001 |
|  | High |  | 565 (68.6%) | 1191 (62.3%) | 377 (56.4%) |  |
| Chips/roast potatoes/croquettes | Low | 3230 | 432 (52.2%) | 944 (49.4%) | 240 (48.5%) | 0.247 |
|  | High |  | 391 (47.5%) | 968 (50.6%) | 255 (51.5%) |  |
| Other potatoes/rice | Low | 3212 | 571 (69.8%) | 1311 (69.0%) | 363 (73.5%) | 0.154 |
|  | High |  | 247 (30.2%) | 589 (31.0%) | 131 (26.5%) |  |
| Hot puddings | Low | 3207 | 676 (82.5%) | 1579 (83.2%) | 409 (83.5%) | 0.887 |
|  | High |  | 143 (17.5%) | 319 (16.8%) | 81 (16.5%) |  |
| Cakes/buns/biscuits/cookies | Low | 3216 | 243 (29.6%) | 541 (28.4%) | 138 (28.2%) | 0.775 |
|  | High |  | 577 (70.4%) | 1365 (71.6%) | 352 (71.8%) |  |
| Salad/coleslaw/raw vegetables | Low | 3215 | 597 (73.0%) | 1419 (74.6%) | 403 (81.6%) | 0.001 |
|  | High |  | 221 (27.0%) | 484 (25.4%) | 91 (18.4%) |  |
| Other cooked vegetables | Low | 3204 | 568 (69.7%) | 1361 (71.8%) | 380 (77.1%) | 0.014 |
|  | High |  | 247 (30.3%) | 535 (28.2%) | 113 (22.9%) |  |
| Yoghurt/fromage frais | Low | 3192 | 681 (83.6%) | 1651 (87.4%) | 424 (87.1%) | 0.027 |
|  | High |  | 134 (16.4%) | 239 (12.9%) | 63 (13.7%) |  |
| Fruit | Low | 3189 | 612 (75.3%) | 1418 (75.1%) | 384 (78.9%) | 0.210 |
|  | High |  | 201 (24.7%) | 471 (24.9%) | 103 (21.1%) |  |
| Mousses/mousse pots/trifles | Low | 3190 | 725 (88.8%) | 1747 (92.7%) | 446 (91.2%) | 0.012 |
|  | High |  | 91 (11.2%) | 138 (7.4%) | 43 (8.8%) |  |
| **Outside school** | | | | | | |
| Chips | Low | 4644 | 963 (81.2%) | 2285 (82.6%) | 552 (79.8%) | 0.180 |
|  | High |  | 223 (18.8%) | 481 (17.4%) | 140 (20.2%) |  |
| Fruit | Low | 4608 | 839 (71.0%) | 2068 (75.6%) | 515 (74.6%) | 0.010 |
|  | High |  | 343 (29.0%) | 668 (24.4%) | 175 (25.4%) |  |
| Burgers | Low | 4620 | 1091 (92.1%) | 2543 (92.6%) | 635 (92.2%) | 0.815 |
|  | High |  | 94 (7.9%) | 203 (7.4%) | 54 (7.8%) |  |
| Sandwiches | Low | 4619 | 919 (77.6%) | 2222 (80.9%) | 546 (79.1%) | 0.051 |
|  | High |  | 265 (22.4%) | 523 (19.1%) | 144 (20.9%) |  |
| Pie/pastie | Low | 4613 | 1094 (92.6%) | 2548 (92.8%) | 642 (93.6%) | 0.720 |
|  | High |  | 87 (7.4%) | 198 (7.2%) | 44 (6.4%) |  |
| Pizza | Low | 4610 | 1042 (88.2%) | 2452 (89.5%) | 604 (87.8%) | 0.324 |
|  | High |  | 139 (11.8%) | 289 (10.5%) | 84 (12.2%) |  |
| Chocolate/sweets | Low | 4965 | 624 (52.0%) | 1490 (53.4%) | 358 (50.8%) | 0.412 |
|  | High |  | 575 (48.0%) | 1301 (46.6%) | 347 (49.2%) |  |
| Crisps | Low | 4646 | 787 (66.1%) | 1884 (68.3%) | 460 (66.0%) | 0.290 |
|  | High |  | 403 (33.9%) | 875 (31.7%) | 237 (34.0%) |  |
| Other | Never | 1847 | 392 (85.8%) | 943 (85.8%) | 254 (87.3%) | 0.798 |
|  | Ever |  | 65 (14.2%) | 156 (14.2%) | 37 (12.7%) |  |

PE, picky eater

Statistical test used was x-tab chi-square test

Total n for each category is different due to participants being able to choose to not answer or skip questions in the Food and Things questionnaire

**Supplementary Table 3**Drinks frequency in the ALSPAC birth cohort study at age 13 years based on longitudinal picky eating classification between ages 2 and 5.5 years (total n=5348)

|  | **Frequency of intake** | **n** | **Picky eating classification** | | | **P value** |
| --- | --- | --- | --- | --- | --- | --- |
|  |  |  |  |  |  |  |
| Pure fruit juice | Low | 5282 | 389 (28.5%) | 896 (28.7%) | 273 (34.5%) | 0.004 |
|  | High |  | 976 (71.5%) | 2230 (71.3%) | 518 (65.5%) |  |
| Squash/Sunny Delight/fruit drinks/Ribena | Low | 5280 | 465 (34.2%) | 1130 (36.1%) | 255 (32.4%) | 0.114 |
|  | High |  | 894 (65.8%) | 2003 (63.9%) | 533 (67.6%) |  |
| Cola drinks | Low | 5286 | 794 (58.3%) | 1851 (59.0%) | 481 (61.0%) | 0.473 |
|  | High |  | 568 (41.7%) | 1284 (41.0%) | 308 (39.0%) |  |
| Other fizzy drinks | Low | 5276 | 820 (60.3%) | 1955 (62.5%) | 505 (63.9%) | 0.197 |
|  | High |  | 540 (39.7%) | 1171 (37.5%) | 285 (36.1%) |  |
| Water or fizzy water on its own | Low | 5228 | 353 (25.9%) | 792 (25.3%) | 214 (27.0%) | 0.594 |
|  | High |  | 1010 (74.1%) | 2341 (74.7%) | 578 (73.0%) |  |
| Flavoured milk drinks | Low | 5287 | 835 (61.4%) | 2039 (65.0%) | 521 (66.0%) | 0.040 |
|  | High |  | 524 (38.6%) | 1100 (35.0%) | 268 (34.0%) |  |
| Low calorie/low sugar soft drinks | Low | 5236 | 1153 (85.8% | 2604 (83.7% | 645 (82.6%) | 0.102 |
|  | High |  | 191 (14.2%) | 507 (16.3%) | 136 (17.4%) |  |
| Decaffeinated cola drinks | Low | 5190 | 775 (58.1%) | 1779 (57.7%) | 402 (52.0%) | 0.010 |
|  | High |  | 560 (41.9%) | 1303 (42.3%) | 371 (48.0%) |  |
| Number of cans/small bottles (up to 500 ml) of soft drinks per week | ≤1 | 5130 | 679 (51.3%) | 1584 (51.9%) | 384 (50.7%) | 0.815 |
|  | ≥2 |  | 644 (48.7%) | 1466 (48.1%) | 373 (49.3%) |  |

**Supplementary Table 4**  Adjusted binary logistic regression analysis of drinks choices of children at 13 years of age by preschool picky eating status

| **Drinks choice** | **Regression analysis** | | | |
| --- | --- | --- | --- | --- |
|  |  | **n** | **OR (95% CI)** | **P value** |
|  |  |  |  |  |
| Pure fruit juice | Low PE | 4742 | 0.97 (0.83, 1.12) | 0.646 |
|  | High PE |  | 0.75 (0.61, 0.91) | 0.004 |
| Squash/Sunny Delight/fruit drinks/Ribena | Low PE | 4742 | 0.95 (0.83, 1.10) | 0.509 |
|  | High PE |  | 1.07 (0.88, 1.31) | 0.477 |
| Cola drinks | Low PE | 4747 | 1.02 (0.89, 1.18) | 0.760 |
|  | High PE |  | 0.95 (0.78, 1.15) | 0.586 |
| Other fizzy drinks | Low PE | 4738 | 0.94 (0.82, 1.08) | 0.402 |
|  | High PE |  | 0.87 (0.72, 1.06) | 0.163 |
| Water or fizzy water on its own | Low PE | 4748 | 1.00 (0.85, 1.17) | 0.945 |
|  | High PE |  | 1.21 (1.48, 1.28) | 0.606 |
| Flavoured milk drinks | Low PE | 4748 | 0.87 (0.76, 1.00) | 0.057 |
|  | High PE |  | 0.88 (0.73, 1.07) | 0.206 |
| Low calorie/low sugar soft drinks | Low PE | 4702 | 1.20 (0.99, 1.46) | 0.067 |
|  | High PE |  | 1.25 (0.97, 1.62) | 0.087 |
| Decaffeinated cola drinks | Low PE | 4658 | 1.03 (0.89, 1.18) | 0.728 |
|  | High PE |  | 1.34 (1.11, 1.62) | 0.002 |
| Number of cans/small bottles (up to 500 ml) of soft drinks per week^a^ | Low PE | 4605 | 1.06 (0.92, 1.22) | 0.409 |
|  | High PE |  | 1.11 (0.91, 1.34) | 0.311 |

Odds ratios (OR) for: Low frequency of intake (reference) (Never/Once a month or less/Once in two weeks) vs High frequency of intake (Once a week/2-3 times per week/4-5 times per week) (or ^a^Low frequency ≤1 per week vs High frequency ≥2 per week).

Adjustments were made for pre-pregnancy body mass index (kg/m^2^), maternal education (none/CSE/vocational, O level/A level or degree) and pre-index pregnancy parity (0 or ≥1) and sex of the child.

n values differ due to missing information on the covariates.

Reference: Never picky eater
